# Convulsions in Children

**Published:** 1903-09-19

**Authors:** 


### Hospital Clinics and Medical Progress.

### CONVULSIONS IN CHILDREN.

The consideration of some points in the causation, prognosis and treatment of convulsions in children forms an instructive lecture by Thomson in the University of Edinburgh.[1] He points out that cases of convulsions in young children are amongst the commonest emergencies to which the medical man in general practice may be suddenly called. The questions most likely to be asked are :—(1) What is to be done while the child is in the fit? (2) Is the convulsion likely to prove fatal? (3) What is it caused by? (4) Is it likely to be followed by other similar attacks? (5) Will the child's future health, and especially his intellect, suffer? (6) What is to be done to ward off further seizures? While it is not possible to answer all these questions until the patient has been under observation for some time, it may be useful to indicate the general principles which serve as guides. With regard to the first point often no special treatment is necessary, though it is customary and sometimes beneficial to put the child in a warm bath for ten or fifteen minutes. Should the seizure last more than five minutes, it is well to administer a little chloroform, a treatment which usually succeeds in quieting the movements and is quite safe ; or chloral hydrate—five to ten grains—may be given to an infant six to twelve months old, either subcutaneously or per rectum if it is not possible to administer the drug by the mouth. The parents may be comforted with the assurance that it is very rare for a baby to die in the first convulsion, though death may occur in the fit from exhaustion, severe intracranial mischief, or laryngeal spasm. It is not always easy to decide upon the cause of the fits in any particular case. Among predisposing causes may be mentioned age, rickets, and inherited nervous constitution, while the determining causes are local disease or injury interfering with the circulation in the brain or its membranes, general morbid conditions, poisons of fevers, etc., and peripheral irritation. The younger the child the more likely is it to suffer from fits of any cause. This liability lessens considerably after two years of age. The predisposition of rickets is well recognised, and the tendency to convulsions rapidly disappears with the improvement of the rickets under treatment. An inherited nervous constitution is another factor to be considered. In children of an excitable temperament convulsive seizures may be provoked by a very slight elevation of temperature or trivial peripheral source of irritation. As regards the determining causes, local brain mischief from injury, abscess, meningitis or tumour, is almost always accompanied by convulsions; engorgement of the vessels of the brain from congenital heart disease or whooping cough may also produce convulsions, and so, too, may cerebral anæmia, such as accompanies severe diarrhœa or hæmorrhage. The general morbid conditions responsible for convulsions are : Sudden rise of temperature, renal insufficiency, many mineral and vegetable poisons. Hence fits are commonly met with at the onset of pneumonia, scarlet fever, measles, erysipelas, influenza, and even chicken-pox. The most frequent forms of peripheral irritation setting up convulsions, especially in children with rickets, are indigestion, constipation, teething or a decaying tooth, middle-ear disease, sometimes adenoid growths, phimosis, and burns. From among the many varieties the following few may be considered as common types of convulsive seizures in infants. In the new-born the occurrence of fits will lead us to suspect brain injury during labour. Cruveilhier found that one-third of all children dying during labour had intracranial hæmorrhage. Over the vertex there may be considerable hæmorrhage without causing death ; in these cases fits frequently ensue during the first week or two. While it is wonderful what injuries new-born children may survive without ill effects, a very considerable proportion of mentally defective children owe their defects to injury at birth. A second group of cases is met with in early childhood, and includes the dyspeptic infant. A third is an important group of cases of convulsions in very young babies, in whom no organic or peripheral cause can be found. These so-called idiopathic convulsions begin during the first month or so, and there is a progressive increase in the number of seizures a day, from two or three to as many as 20 or 30 attacks in the 24 hours, and this may continue for weeks, unless active measures are employed. There are no other symptoms of brain mischief, and no abnormalities whatever can be found. Bromides and treatment of the alimentary canal are useless in these idiopathic cases. What is necessary is to put the child under the influence of chloral hydrate as soon as possible. One or two grains every two hours is not too large a dose given by the mouth. Usually in three or four days the drug can be discontinued without any return of the convulsions, though the effect of the medication, drowsiness, may last some time.

The commonest cases of convulsions are those associated with rickets and teething. They are usually met with between six months and two years ; more often in the cold weather, especially in early spring. The signs of rickets are frequently not well marked, but are rather those of the early stages of the disease. These cases are often associated with facial irritability, laryngismus, or tetany, and the treatment is directed to regulation of the diet on anti-rachitic lines. In the last group of cases the convulsions are one and perhaps the only sign of serious brain mischief, and are the ones which are apt to give place to regular epileptic seizures. Enough has been said to indicate the general principles of classification of the causes of convulsions. Diagnosis will depend upon a thorough examination, with the infant stripped, and little can be told until all data have been collected. It is only by watching the course of the case under treatment that the prognosis can be safely stated. As regards the treatment in a particular case everything will depend on the determination of the cause, hence the importance and interest which centres about the proper recognition of the many infantile diseases accompanied by convulsions.

[1] International Clinics, Vol. I., Series 13, page 169.

